# Room Temperature Consolidation of a Porous Poly(lactic-co-glycolic acid) Matrix by the Addition of Maltose to the Water-in-Oil Emulsion

**DOI:** 10.3390/ma9060420

**Published:** 2016-05-27

**Authors:** Eliana Esposito, Flavia Ruggiero, Raffaele Vecchione, Paolo Antonio Netti

**Affiliations:** 1 Center for Advanced Biomaterials for Healthcare, Istituto Italiano di Tecnologia (IIT@CRIB), Largo Barsanti e Matteucci, Napoli 5380125, Italy; eliana.esposito@iit.it (E.E.); flavia.ruggiero@iit.it (F.R.); paolo.netti@iit.it (P.A.N.); 2 Interdisciplinary Research Center on Biomaterials, (CRIB), University of Naples Federico II, Naples 80125, Italy; 3 Department of Chemical, Materials & Industrial Production Engineering, University of Naples Federico II, Naples 80125, Italy

**Keywords:** PLGA, maltose, porous matrix, polymer microneedles, electro-drawing

## Abstract

In composite materials made of polymer matrices and micro-nano dispersed compartments, the morphology of the dispersed phase can strongly affect several features of the final material, including stability, loading efficiency, and kinetic release of the embedded molecules. Such a polymer matrix composite can be obtained through the consolidation of the continuous polymer phase of a water-in-oil (W/O) emulsion. Here, we show that the morphology of the dispersed phase in a poly(lactic-co-glycolic acid, PLGA) matrix can be optimized by combining an effective mild temperature drying process with the addition of maltose as a densifying compound for the water phase of the emulsion. The influence of this addition on final stability and consequent optimal pore distribution was theoretically and experimentally confirmed. Samples were analyzed in terms of morphology on dried flat substrates and in terms of rheology and interfacial tension at the liquid state. While an increase of interfacial tension was found following the addition of maltose, the lower difference in density between the two emulsion phases coming from the addition of maltose allowed us to estimate a reduced creaming tendency confirmed by the experimental observations. Rheological measurements also confirmed an improved elastic behavior for the maltose-containing emulsion.

## 1. Introduction

In the last several decades, polymers have been increasingly used for drug delivery in different applications such as tumor therapy [1] and immune-therapies [2]. Polymer-encapsulated drugs are in general more effective than their freely delivered counterparts, since polymer-loaded drugs are protected from degradation [3]. This protection provides a longer biological half-life and a potentially improved efficacy with reduced systemic side effects. This stabilization also applies to proteins. For example, polymeric microspheres encapsulating proteins have been proved to be effective in conveying and releasing even very labile bioactive moieties in a specific manner at pre-programmed rates [4,5,6]. These systems effectively protect their “protein cargo” from inactivation occurring in biological environments and preserve its bioactivity during the release process [7]. Among the various materials, PLGA, a biocompatible member of the aliphatic polyester family of biodegradable polymers, is one of the most used, being approved by Food and Drug Administration [8]. It has been used to embed even very labile proteins such as vascular endothelial growth factor (VEGF), a potent angiogenic molecule [9]. Due to the low affinity of hydrophilic molecules, VEGF was embedded in the porous structure of the polymer at the time of preparation. Alternatively, the loading of hydrophilic molecules can be carried out after the preparation of a porous structure, limiting this approach to open and interconnecting pores [10]. 

Macroporous polymers are typically produced using sacrificial porogens [11], particle templating [12], freeze-drying applied to aerogels [13], or emulsions, which enables the improvement of the solubility of poorly soluble drugs [14]. Emulsion templating is a flexible and easily controlled method for the fabrication of porous materials. The principle of fabrication is quite simple: it consists of a block structure of continuous phase by polymerization or freezing, followed by removal of the internal phase. Generally, emulsions have an average droplet size of at least several micrometers, and the droplets have a rather broad size distribution, unless special procedures are adopted. The porosity of a matrix strictly depends on the composition of the starting emulsion; indeed, if a high amount of internal phase is present, >74% *v*/*v*, a well interconnected porosity can be obtained, whereas, if a less concentrated emulsion is used (internal phase volume <60%), pores will generally have a closed-cell structure [15]. By changing the content of the dispersed phase, it is possible to create very different structures, which have been employed in various applications, such as matrix adsorbents for carbon dioxide [16], scaffolds for biological tissues [17,18,19], and carriers for drug delivery, such as microneedles for transdermal delivery [20,21]. Consolidation of the porous structure in the case of emulsions can occur through solvent extraction from the continuous phase. As emulsions are instable systems, they tend to phase-separate before consolidation, especially for high concentrations of the dispersed phase. This way, the morphology of the inner phase cannot be controlled, leading to uncontrollable release properties. A fast thermal consolidation may fix the morphology, but it could damage thermo-labile embedded molecules. 

Nanoprecipitation has also been proposed as a valid alternative to the typical instability of water-in-oil (W/O) emulsions to encapsulate high payloads of hydrophilic molecules, as in the case of cucurbitacin I loaded in PLGA [22]. However, this method requires an external water phase for the extraction of the solvent of the inner oil phase, so it does not apply to systems where the oil phase is the external one. Recently, at least in the case of O/W emulsions, the addition of maltose in the water phase has been proposed as an effective way to enhance stability [23]. It was correlated to the increase of the emulsion continuous phase viscosity. Concerning the W/O emulsions, how the addition of electrolytes to the water phase can determine an improved stabilization of the system has been demonstrated [24], as it acts on Laplace pressure. Other kinds of additives for the water phase, such as proteins or sugars (mainly focusing on glucose), have also been tested in order to improve the emulsion stability by means of osmotic effects [25]. However, theoretical treatises of the stabilizing mechanism as well as the application of maltose to the stabilization of W/O emulsions have not been reported so far to the best of our knowledge. On the other hand, maltose has been previously introduced in the literature as effective material for the fabrication of dissolving drug delivery systems, such as microneedles [26].

Here, we propose an alternative to the fast thermal consolidation based on the modification of the dispersed phase by the addition of maltose as a dissolving molecule, which improved the stabilization of the emulsion, thus allowing a slow consolidation at room temperature. We carried out a rheological characterization of the emulsion in order to confirm the improved stability feature. Finally, we assessed that this strategy can be applied to the electro-drawing technology for the preparation of porous biodegradable PLGA microneedles [27].

## 2. Experimental Section

### 2.1. Materials

For the preparation of the porous polymeric matrix, we used poly(lactic-co-glycolic acid, 50:50) (C.A.S. 26780-50-7), (PLGA) Resomer RG 504H supplied by Evonik industries (Essen, Germany). Dimethyl carbonate (DMC) (C.A.S. 616-38-6) used as solvent, maltose monohydrate (C.A.S. 6363-53-7) used as the additive in the water phase, and albumin, tetramethylrhodamine isothiocyanate bovine (TRITC-albumin), used as model drug, were purchased from Sigma Aldrich (St. Louis, MO, USA). Lecithin was used as a biocompatible surfactant to stabilize emulsion and was purchased from Lipoid (Ludwigshafen, Germany).

### 2.2. Samples Preparation

PLGA was dissolved in DMC as a non-toxic and environmentally friendly solvent at 25% *w*/*v*. It represents the continuous phase of the W/O emulsions, kept constant for all samples prepared for the experiments. For the first set of samples, 180 mg/mL of lecithin were dissolved in water; emulsions were set up with different amounts of water—30%, 60%, 80%, and 100% in weight with respect to PLGA. These water phases were emulsified by using an immersion sonicator (Ultrasonic Processor VCX500, Sonic and Materials, Newtown, CT, USA) for 20 s at 30% power, keeping samples in an ice bath to avoid water evaporation. The consolidation of the polymeric matrix was carried out in a specific mold to standardize the results, making them independent from the surface exposure. Molds were obtained by putting a punched PDMS layer, with 6-mm diameter holes, on a microscope glass slide. 15 µL of emulsion were injected into each well and prepared for drying. The first set of samples was consolidated following two procedures: the first at 50 °C and the second at room temperature, both for 24 h. By doing so, solvent evaporation occurred very quickly in the first case and much more slowly at room temperature.

For the second set of samples, water content in the emulsion was fixed at 80%; half of the samples were prepared with water and lecithin as in the first case, while in the second case they were prepared by adding maltose until saturation (corresponding to 360 mg/mL in the inner water phase). Emulsification and preparation of the samples through the use of molds was carried out following the procedures previously described. This time consolidation was carried out at 30 °C or at 30 °C in a vacuum (~100 mbar) to increase the rate of solvent evaporation. Process parameters for each preparation are summarized in Table 1.

### 2.3. Emulsion Stability

In order to investigate the stability of the emulsions in liquid state introduced by solvent evaporation without alterations, a Turbiscan LAB was used. This instrument measures backscattering and transmission in respect to the sample height in order to detect particle size change (coalescence, flocculation) and phase separation (sedimentation, creaming). We prepared 20 mL of emulsions, with and without maltose in water phase, and backscattering was measured every hour for 24 h.

### 2.4. Morphological Analysis of Porosity

Sections of matrices were prepared using Leica CryoUltra Microtome EM-FC7-UC7. Thin strips were incorporated in polydimethylsiloxane (PDMS), cured for 24 h at room temperature, and then frozen at −140 °C. Finally, they were sectioned at a thickness of 5 µm. Pictures of porosity were taken using field emission SEM (Ultra plus Zeiss, Jena, Germany), after the sputter coating of the samples with a 15-nm-thick gold layer, imposing 5 kV of voltage (EHT). Image analysis was carried out by means of the software Fiji (V. 1.50b, Wayne Rasband, National Institutes of Health, Bethesda, MD, USA) in order to calculate porosity percentage and pore diameter. Pores were identified with the help of an object counter, which quantifies areas for each object. Since, with an acceptable approximation, the pore shape can be considered circular, we are able to directly calculate the diameter of the pores. To estimate porosity, the areas of all pores were summed and related to the entire area of sample (Equation (1)):

(1) $$\text{Porosity}\;\left(\%\right)=\left(\;\frac{\sum \text{​}A_{\text{pore}}}{A_{\text{sample}}}\right)\times 100,$$

where *A*_pore_ is the value of the area of each pore as quantified by the object counter, and *A*_sample_ is the area of the entire sample known through the dimensions of the image acquired. To analyze the dimensional distributions of the pores and quantify porosity (%), three samples for each type of material and three sections for each sample were considered. In particular, for the dimensional distributions of the pores, 100 pores for each sample were measured.

### 2.5. Interfacial Tension Measurements

Interfacial tension measurements between the PLGA/DMC continuous phase and the two different dispersed phases made up by adding lecithin to both pure water and maltose containing water, respectively, with the composition indicated in the Materials section, were carried out by using an optical tensiometer (Attension Theta Lite, Biolin Scientific, Stockholm, Sweden) and the reverse pendant drop method. After the stabilization of their shape under the surfactant action, drop images were recorded and analyzed by means of the Attension Theta software. The Young–Laplace curve fitting method was employed. The measurements were repeated at least three times, each time on a sample made of ten drops. The results are presented in Section 3 as mean values, coupled to their standard deviations.

### 2.6. Rheological Measurements

The viscoelastic properties of PLGA emulsions with 80% dispersed phase, both with and without maltose, were measured by means of a stress-controlled rheometer (MCR 302 rheometer, Anton Paar, Graz, Austria), fitted with a double-gap concentric-cylinder geometry (DG 27, Anton Paar, Graz, Austria) in order to avoid DMC evaporation during the measurements. Temperature was kept at 25 °C with an accuracy of ±0.1 °C during the measurements by an Anton Paar Peltier temperature device for concentric-cylinder systems, equipped with a water circulating bath. All samples were placed in the double-gap cylinder measuring system and left to rest for 2 min for structure recovery and temperature equilibration. Dynamic oscillatory shear measurements were carried out for the frequency sweep tests. The viscoelastic linear region was preliminarily determined by means of amplitude sweep tests (0.01%–100% strain, 0.1 Hz, data not shown). From frequency sweeps, ranging from 1 to 0.01 Hz at a strain of 10%, viscoelastic parameters of the emulsions, such as storage (G´) and loss (G´´) moduli, were determined. All measurements were repeated three times on each sample. The data are plotted as mean values in Section 3. Their standard deviations are not plotted in the graph, as they are lower than 10%.

### 2.7. Microneedles Fabrication

Microneedles were fabricated following an electro-drawing (ED) process previously described in the literature [27]. Starting solutions are emulsions containing 80% water phase, with and without maltose. Briefly, emulsion drops were deposited on PDMS pillars integrated on a flexible substrate and then positioned under a lithium tantalate crystal, which was locally heated in correspondence with the drops in order to draw them and create the cone-like shape. After the electro-drawing process, microneedles were kept at 30 °C for 2 h in a low vacuum (100 mbar) to accelerate solvent evaporation and fix their shape. We are currently working on an alternative way to preserve the shape by modifying the ED process flow so that no vacuum is required at all. Finally, the microneedles were stored in dry conditions with silica gel until characterization tests to avoid moisture absorption.

### 2.8. Indentation Test

To verify the mechanical resistance of the microneedles, they were tested through indentation in paraffin wax. The support of the microneedles was fixed onto a motorized translation stage (Thorlabs MTS25-Z8); indentation velocity was standardized at 2 mm/s.

## 3. Results and Discussion

### 3.1. Analysis of PLGA Matrix Porosity

**Figure 1:**
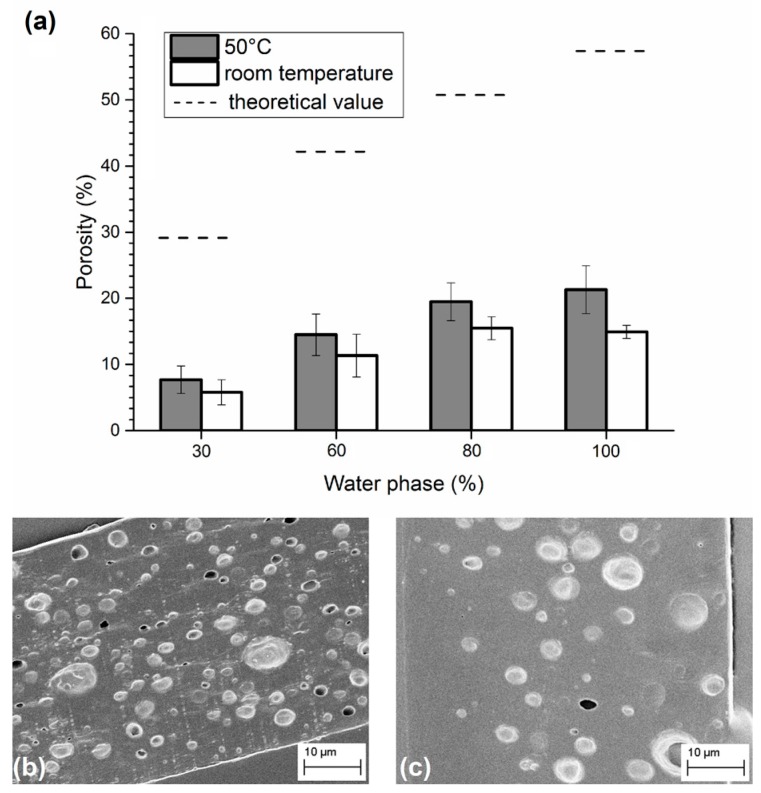
(**a**) Porosity obtained in emulsions containing different amounts of water phase and consolidated at 50 °C or at room temperature. SEM images of sliced matrices obtained from emulsions with 80% of water phase and solidified at (**b**) 50 °C; and (**c**) at room temperature.

The first set of emulsions was used to compare the effects of the solvent evaporation speed. As shown in Figure 1, emulsions treated with a higher temperature preserved a higher level of porosity with respect to samples consolidated at room temperature, as the solvent evaporation, *i.e.*, the matrix consolidation, is faster in the latter case. Indeed, if the solvent evaporates very quickly, water drops have less time to move within the matrix. The final mean porosity, limited to the low content of the dispersed phase, was directly proportional to the water added to the emulsion until reaching a plateau at 80% *v*/*w*, where the porosity level was ~20%. In the case of slow evaporation, the porosity level was ~14%, and the plateau was reached even earlier (60% *v*/*w*). Although a fast evaporation yields better results, the obtained level of porosity was still lower than half of the theoretical value (dashed lines in the figure) calculated considering the amount of water in the emulsion and the volume of polymers. These results are probably explainable with a separation phase that occurs during the consolidation process, thus causing a loss of porosity, which depends on the evaporation rate: the lower the rate, the higher the loss.

**Figure 2:**
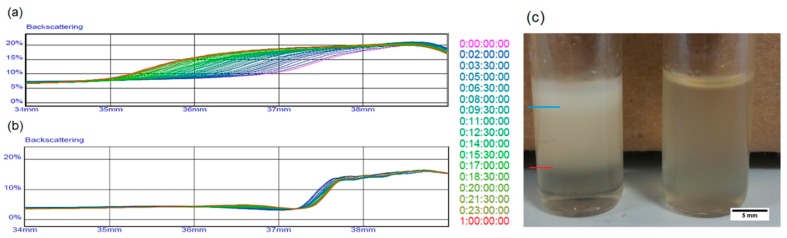
Stability comparison of emulsions in liquid state through backscattering analysis of (**a**) the sample without maltose and (**b**) the sample with maltose; (**c**) Comparison between emulsions one week after their preparation. The blue line in the figure indicates the level of water separated from the emulsion, while the red line marks a region deprived of water microdroplets.

This loss of porosity represents a big issue in drug delivery applications since it reduces the amount of drug that can be loaded inside the polymeric matrix. The final porosity should be as close as possible to the theoretical value. To limit phenomena of coalescence and phase separation, we experimented with water phase densification by adding maltose. Figure 2 shows the behavior of the two different emulsions. In particular, backscattering analysis underlines creaming phenomena in the emulsion without maltose, showing stratification of water phase in the upper part of the sample, while the emulsion containing maltose remains stable. One week after preparation (Figure 2c), the effect of destabilization was well visible to the eye.

**Figure 3:**
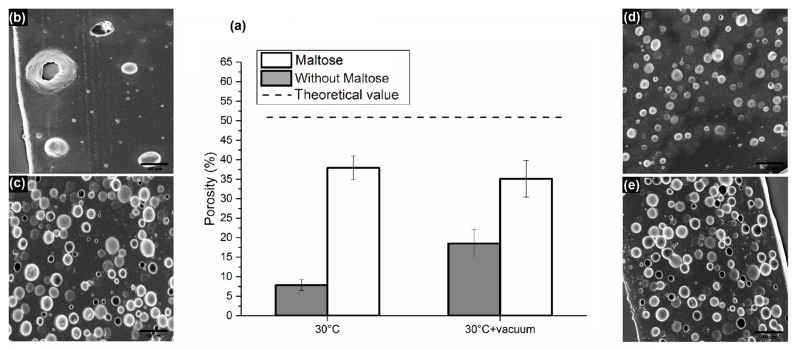

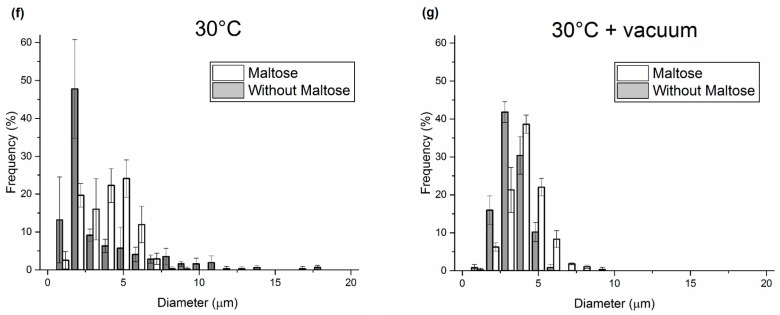
(**a**) Comparison of porosity obtained from emulsions with and without maltose in water phase, consolidated at 30 °C and 30 °C in a vacuum; SEM images of sliced matrices obtained from emulsions consolidated at 30 °C (**b**) without maltose; and (**c**) with maltose in water phase; SEM images of sliced matrices obtained from emulsions consolidated at 30°C with vacuum—(**d**) without maltose; and (**e**) with maltose. (Bottom) Dimensional pore distributions of the above-mentioned samples consolidated at (**f**) 30 °C; and (**g**) 30 °C with a vacuum.

In a second set of samples, water content was fixed at 80% to make the comparison between samples with and without maltose possible, since increasing water to 100% did not entail an improvement in porosity in the case of the absence of maltose. The consolidation process was carried out at 30 °C only or at 30 °C in a vacuum. We chose to work at a mild temperature in order to guarantee safety conditions to thermo-labile biomolecules preloaded in the matrix. In particular, we compared samples with and without maltose after consolidation at 30 °C; SEM images showed a porosity, which, in the presence of maltose and in the absence of a vacuum, was about seven times higher than samples with no maltose (Figure 3). Particularly, despite an evaporation time, which is much longer than in the case of dehydration at 50 °C, maltose seems to limit the drop aggregation and phase separation, bringing the level of porosity between 30% and 40%. Together with the decisive influence of maltose, the contribution of a vacuum during consolidation was also analyzed. As can be seen in Figure 3f,g, there is a significant improvement in the distribution of the pores in the case of samples dried in a vacuum, even if there is not a great difference in the level of porosity for emulsions containing maltose. A vacuum accelerates solvent extraction, requiring less time to drop coalescence before the immobilization of porosity. A 3D analysis of the pores by loading the water phase with TRITC-albumin and carrying out a confocal analysis was also performed and reported in the Supplementary Materials (Figures S1–S3).

### 3.2. Interfacial Tension and Rheological Analysis of the Emulsions

In order to elucidate the reasons at the basis of the experimentally observed improved stability of the PLGA emulsion containing maltose, the densities and the interfacial properties of the water phases in the PLGA/DMC solution were evaluated. The obtained results are summarized in Table 2.

First, the interfacial tensions of the water phases without surfactant within the PLGA/DMC polymer solution were measured: the higher value obtained for the water/maltose system suggests that the emulsion formed by loading maltose in the water phase should be more instable with respect to the emulsion without maltose. This latter evidence meant that a higher level of energy was needed in order to expand the interface of the dispersed phase of a unit length. The addition of lecithin as a surfactant determined a lowering of the interfacial tension in both cases, as expected; however, again, the pure water system was shown to be more stabilized than the water/maltose one. This increase in interfacial tension recorded after the introduction of maltose as an additive is in agreement with the results reported in [25] for similar kinds of additives.

This conclusion appears in opposition with the observed longer time stability of the emulsion in the presence of maltose. Indeed, the higher interfacial tension for the maltose-containing system could determine a larger size of the dispersed droplets because of the higher level of energy required for interface stabilization: it is well known that, the larger the dispersed drops are, the higher their tendency to cream is, with a consequent lowering of the emulsion stabilization degree. Conversely, from the calculation of the density values of the phases, it appeared clear that the maltose-containing emulsion was characterized by a lower difference in density between the continuous and the dispersed phase, leading to a reduction in the creaming tendency. From the experimental observation confirming a reduced creaming tendency in the presence of maltose, it is possible to conclude that the density effect was strong enough to counteract the higher creaming tendency due to the bigger droplet size with respect to the system containing no maltose. 

Theoretically, the settling or creaming velocity *U* of droplets within an emulsion can be estimated by means of the following relation:

(2) $$U=\frac{2a^{2}\left|∆\rho \right|g}{9\mu }$$

where *a*, ∆ρ, g, and μ are respectively the droplet radius, the difference in density between the dispersed and the dispersing phases, the gravitational constant, and the continuous phase viscosity. Therefore, in constancy of the continuous phase viscosity, the settling rate is proportional to the product:

ɑ^2^ |∆ρ| (3)

As a confirmation of the observed higher creaming rate of the emulsion without maltose, by assuming the mean values of the distributions in Figure 3 as radii for the droplets and the values in Table 2 as the densities, from Equation (3), it is possible to obtain

1. emulsion containing no maltose: (1.5 × 10^−6^)^2^ × (1150 − 964) = 4.2 × 10^−10^ Kg/m; and
2. emulsion containing maltose: (2 × 10^−6^)^2^ × (1150 − 1098) = 2.1 × 10^−10^ Kg/m,

*i.e.*, a reduction of about 50% of the creaming rate.

Therefore, also theoretically, it is confirmed that the maltose introduction contributes to the kinetic stability of the PLGA emulsion.

**Figure 4:**
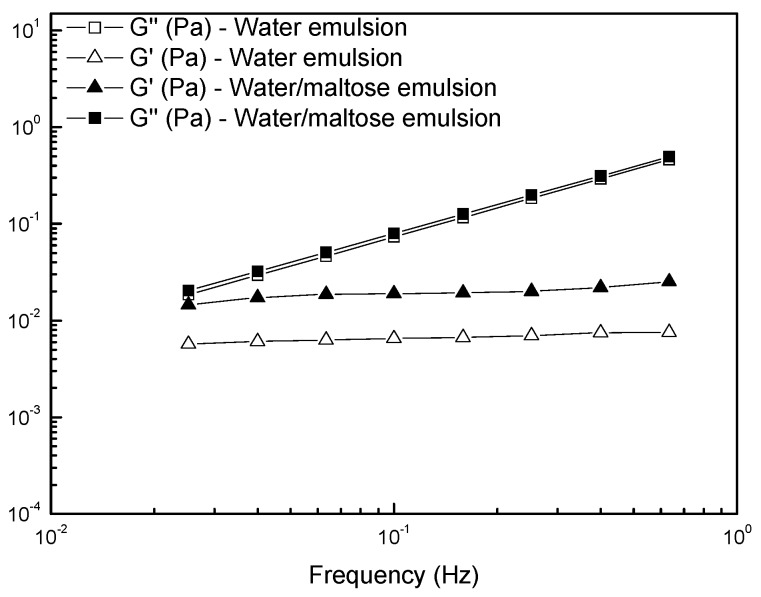
Frequency dependence of the storage modulus G’ and loss modulus G’’ for the studied emulsions.

In Figure 4, we reported the results of the frequency sweeps carried out on the emulsions in terms of storage (G’) and loss (G’’) moduli as functions of the oscillations frequency. In almost the whole range of investigated frequencies, the samples showed a viscous liquid-like behavior (G’’ > G’) because of the low volume fraction of the dispersed phase. The loss modulus trend was almost the same for the two studied samples, the viscous component being mainly due to the continuous phase. For the storage modulus, a higher value for the emulsion containing maltose was found: the elastic component, due to the surface contribution arising from the dispersed phase, was higher in the presence of maltose dissolved in the dispersed domains. This latter evidence is in agreement with the lower tendency of the maltose-containing system to kinetic destabilization by means of coalescence, which would increase the size of the drops with a further acceleration of the creaming process.

### 3.3. Application of the Proposed Method in the Field of Porous Polymer Microneedles

**Figure 5:**
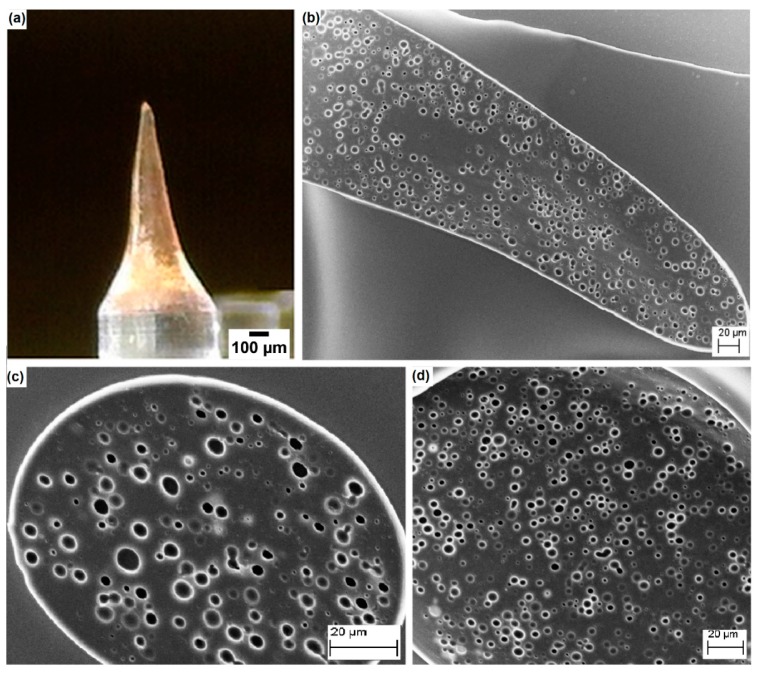
(**a**) Electro-drawn microneedle laying on a PDMS pillar. SEM images of (**b**) a longitudinal section; (**c**) transversal sections near the tip; and (**d**) near the base of the microneedle. Needle structure shows the same porosity of a flat layer homogeneously distributed in all its parts.

Finally, we tested the employment of emulsions, finalized to the formation of microneedles by the electro-drawing process. The electro-drawing is a mask-less and mold-less 3D lithography process in which the microneedles are fabricated under the action of the electro-hydrodynamic pressure induced by a pyroelectric effect (pyro-EHD). A microneedle is shown in Figure 5. By using the emulsion with the addition of maltose and a consolidation procedure at 30 °C in a vacuum, we were able to obtain microneedles with good porosity, evenly distributed throughout the length of the cone, which represents an improvement as compared to the case in the absence of maltose [27]. Interestingly, the morphology of the pores in the electro-drawn emulsions was similar to that of previous samples consolidated without electro-drawing.

**Figure 6:**
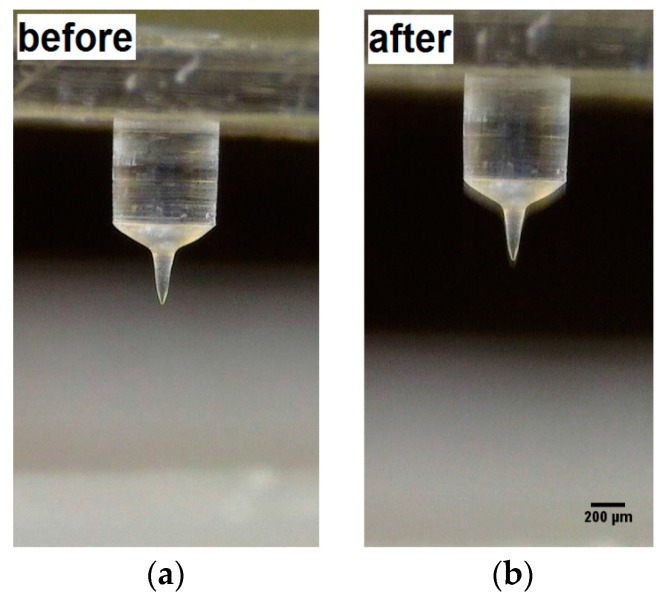
Microneedle obtained by electro-drawing of emulsion containing maltose (**a**) before; and (**b**) after indentation in paraffin wax.

As proof of the mechanical strength of the as-prepared porous microneedles containing maltose, an indentation in paraffin wax was performed. No damage was evidenced after the indentation process, as shown in Figure 6 and in the Supporting movie.

## 4. Conclusions

Microporous PLGA matrices were prepared by emulsification of a water phase in a solution of PLGA. To keep good uniformity of the final pores typically prevented by the instability issue, we introduced a method based on the modification of the dispersed phase by the addition of a dissolving molecule: maltose. Maltose improved the stabilization of the emulsion allowing its consolidation at a very mild temperature (30 °C) and in the absence of a vacuum. We assessed the improved performance of the system by evidencing the higher quantity and homogeneous distribution of the final pores. We explained this behavior in terms of balance between the density of the water phase containing maltose and the dispersing phase characterized by the PLGA solution by using the creaming velocity formula. We also made some rheological considerations on the storage modulus, which was found to be higher in the case of systems with maltose, justifying a lower tendency to coalescence and therefore to creaming. Very interestingly, we assessed that this strategy can be applied to the electro-drawing technology for the preparation of porous biodegradable PLGA microneedles keeping the optimized pore morphology perfected in flat matrices.

## Figures and Tables

**Table 1:** Summary of process parameters for each preparation.

Sample Set 1: Water Phase Composition 180 mg/mL Lecithin		Sample Set 2: Water Phase Content 80%	
Water Content	Consolidation Process	Water Phase Composition	Consolidation Process
30%	Room temperature	180 mg/mL lecithin	30 °C
30%	50 °C		
60%	Room temperature	180 mg/mL lecithin	30 °C + vacuum
60%	50 °C		
80%	Room temperature	180 mg/mL lecithin and 360 mg/mL maltose	30 °C
80%	50 °C		
100%	Room temperature	180 mg/mL lecithin and 360 mg/mL maltose	30 °C + vacuum
100%	50 °C		

**Table 2:** Density of the phases of the studied emulsions; interfacial tensions between the studied dispersed phases and the PLGA/DMC solution.

Phase	Density (g/mL)	Interfacial Tension (mN/m)
PLGA/DMC 25% *w*/*v*	1.15	-
Pure water	1	7.2 ± 0.25
Water/maltose	1.13	8.5 ± 0.38
Water/lecithin	0.964	4.7 ± 0.3
Water/lecithin/maltose	1.098	7.3 ± 0.6

## Supplementary Materials

The following are available online at www.mdpi.com/1996-1944/9/6/420/s1.
