# Supplementary material for: Room Temperature Consolidation of a Porous Poly(lactic-co-glycolic acid) Matrix by the Addition of Maltose to the Water-in-Oil Emulsion
Source: Materials (Basel). 2016 May 27;9(6):420. doi: 10.3390/ma9060420 (PMC5456783; doi:10.3390/ma9060420)
Supplement: Supplementary file 1 [file materials-09-00420-s001.pdf]

Supplementary Materials: Room Temperature Consolidation of a Porous Poly(lactic-co-glycolic acid) Matrix by the Addition of Maltose to the Water-in-Oil Emulsion

Eliana Esposito, Flavia Ruggiero, Raffaele Vecchione and Paolo Antonio Netti

Fluorescence Analysis of Porous Matrix

A confocal analysis of the porosity, was performed by making fluorescent the pores. To this aim, TRITC-albumin, commonly used to mimic behaviour of hydrophilic drugs with high molecular weight, was dissolved in water phase at 1 μM and sonicated for few minutes to break aggregates. Samples preparation was carried out following the same procedure described in Paragraph 2.2 (*Sample preparation*) with 80% of water content in the emulsion. Fluorescence analysis was performed in 3D using confocal microscope Leica TCS at 543 nm equipped with a 25× water immersion microscope objective. Z-stack acquisitions, were conducted collecting images each 1.5 μm which is the slice thickness of the images. From the sequence of images, showing the material at different height levels, it is clear the presence of irregular and larger pores in samples without maltose and consolidated at 30 °C (Figure S1) as compared to samples obtained in presence of maltose where the distribution of pores is uniform and homogeneous overall the sample volume (Figure S2).

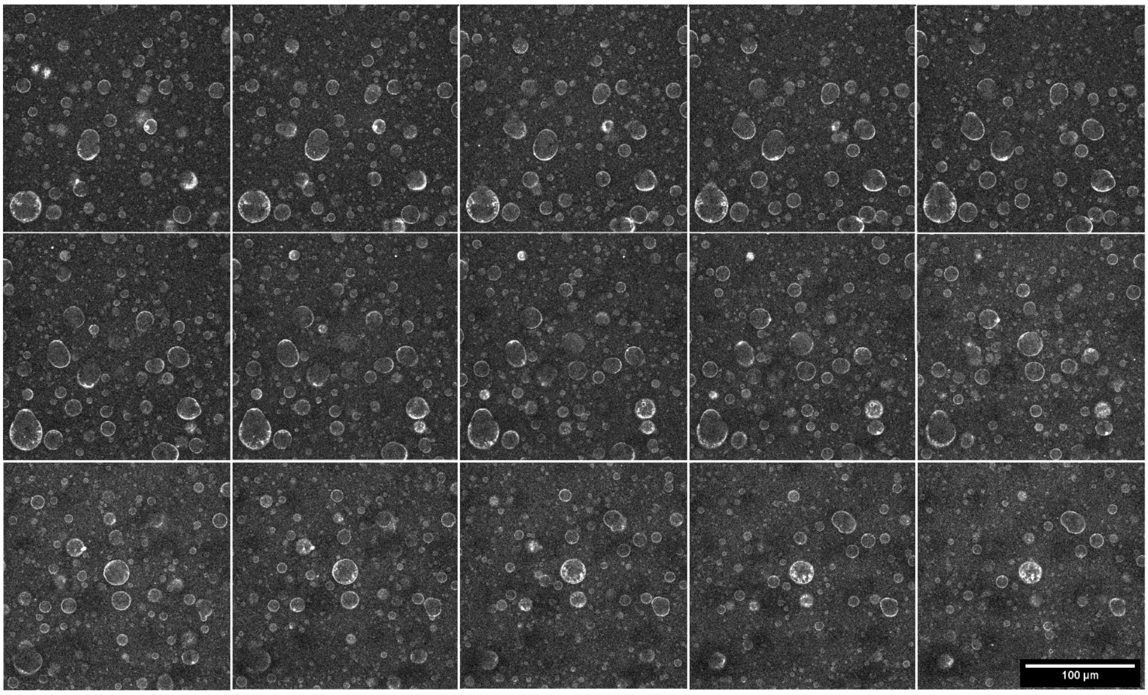

Figure S1. Z-stack of material obtained from the emulsion with no maltose and consolidated at 30 °C.

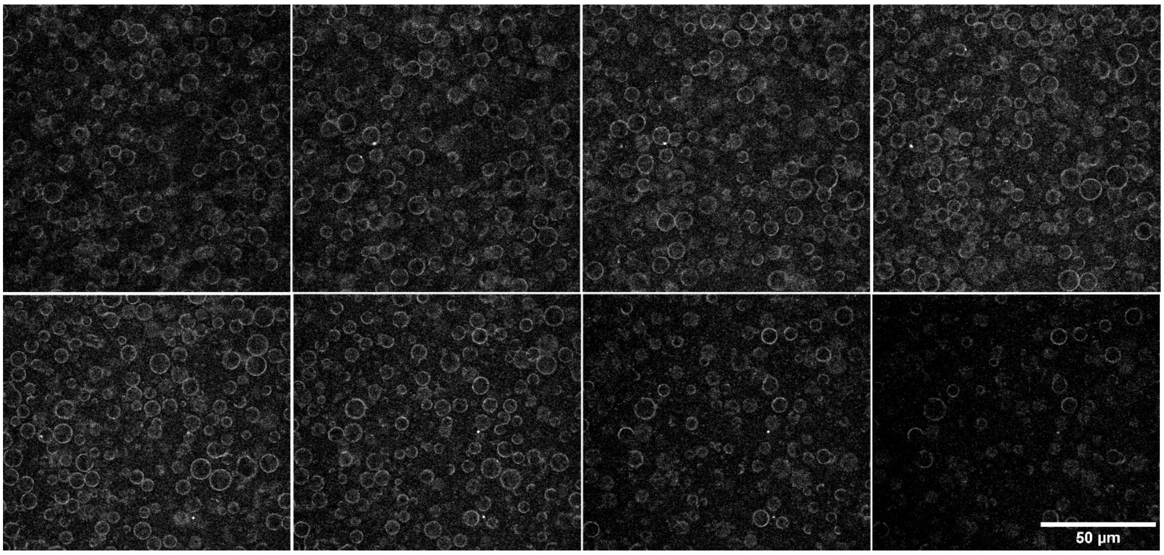

Figure S2. Z-stack of the material obtained from the emulsion containing maltose and consolidated at 30 °C.

Samples obtained in duplicate from emulsions with and without maltose and consolidated at 30 °C were weighed at different times to monitor solvent evaporation. By measuring the weight of the samples consolidated at 30 °C without vacuum, we noticed that both matrices with and without maltose lost about 70% of their initial mass (Figure S3). After 1 h, samples mass was quite stable at ~25% of initial value, so we can assume the morphology of matrix already blocked after this time. This means that any difference in the final morphology is associated to the very first minutes.

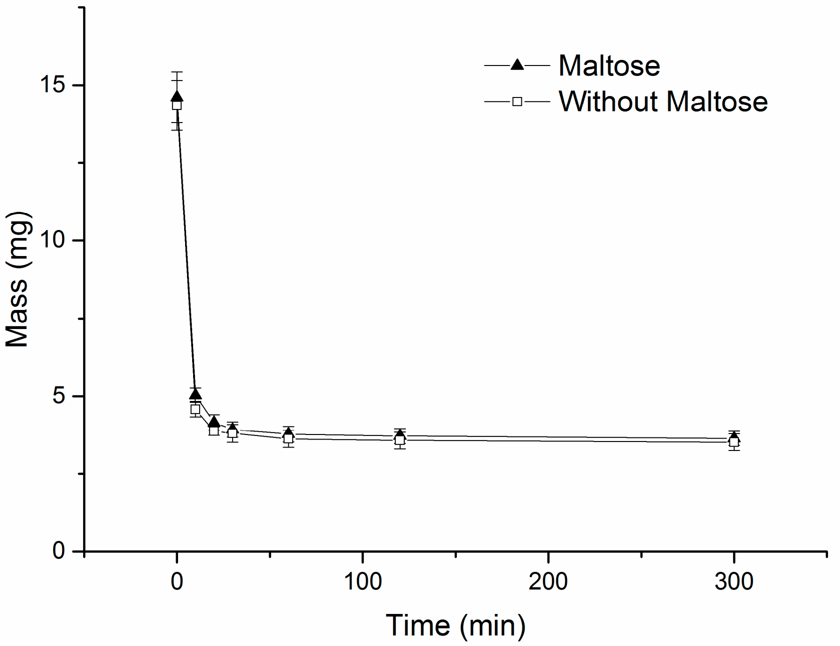

Figure S3. Weight loss analysis in samples consolidated at 30 °C without vacuum.
